# Volume Matters: Dilution of Soil Inoculum Reduces Positive Plant–Soil Feedback in *Pinus radiata* Seedlings

**DOI:** 10.3390/plants15050809

**Published:** 2026-03-06

**Authors:** Joanna L. Green, Lauren P. Waller, Christel Brunschwig, Simeon Smaill, Leo Condron

**Affiliations:** 1 Agriculture and Life Sciences, Te Whare Wānaka o Aoraki, Lincoln University, Lincoln 7647, New Zealand; 2 Bioeconomy Science Institute, Titokorangi Drive, Private Bag 3020, Rotorua 3046, New Zealand; christel.brunschwig@scionresearch.com; 3 Bioeconomy Science Institute, Tuhiraki, 19 Ellemere Junction Road, Lincoln 7608, New Zealand; 4 Ngāi Tahu Forestry, P.O. Box 13 046, Christchurch 8141, New Zealand

**Keywords:** plant–soil feedback (PSF), positive feedback, dilution effect, inoculum volume, soil legacy, seedling establishment, pine seedlings, *Pinus radiata*

## Abstract

Soil conditioning can generate persistent plant–soil feedbacks (PSF) that influence plant performance under subsequent growth conditions, yet the role of soil inoculum volume in mediating these effects remains poorly understood. Here, we tested how inoculum volume influences the relative strength of a known positive PSF effect. We performed a plant–soil feedback experiment with *Pinus radiata* D. Don in two phases: one, a “conditioning phase”, and two, a “feedback phase”, where inoculum from the first phase was used in different dilutions to test the growth differences resulting from conditioning. To understand how inoculum volume affects subsequent growth in the feedback phase, seedlings (*n* = 12 per treatment) were grown in soil from phase one using different volumetric dilutions; 100% conditioned soil, 50% conditioned soil + 50% inert media, or 25% conditioned soil + 75% inert media. Positive plant–soil feedbacks were observed in undiluted soils: seedlings produced 40–65% greater biomass and experienced 50–70% lower mortality compared to the lowest inoculum treatment. However, this response varied with dilution; the strength of plant–soil feedbacks decreased with increasing dilution of inoculum. These findings highlight soil inoculum volume as an important, but often overlooked, factor in plant–soil feedback experiments and applied soil management. Our study provides experimental evidence that effective soil conditioning depends on both conditioning and a required minimum inoculum volume to confer measurable benefits to future plantings.

## 1. Introduction

Soil conditioning experiments, such as plant–soil feedback (PSF) test how plants modify the biotic and abiotic properties of soil in ways that influence the performance of subsequent plants growing in those soils [1,2]. Feedbacks can be positive or negative and are increasingly recognized as important drivers of plant growth, competition, and ecosystem responses to environmental change. In particular, soils can retain a legacy or “memory” of past environmental conditions, which alters soil microbial communities and can influence plant responses to future stress events [3,4]. Understanding how these soil legacies affect plant performance is especially relevant under predicted climate scenarios of increased drought frequency and intensity.

Plant–soil feedback experiments often demonstrate that plant performance is strongly affected by previous occupants of the soil, whether they be heterospecifics [5,6], conspecifics [5,7,8] or communities [5,9]. These effects are often attributed to changes in soil microbial communities that are better adapted to specific hosts or environmental regimes [10]. Pine trees, as an ectomycorrhizal species, have been found to leave a beneficial legacy for other pines, often in greater proportions of fungal associates [8,11,12]. Such systems provide an opportunity to test not only the presence of positive conspecific feedback, but also the mechanisms that determine their persistence and strength.

The strength and consistency of PSFs vary widely among studies for all different types of plants [2,13] and for trees specifically [14,15,16], and practical questions about when and how these effects exist remain. One critical but underexplored factor is soil inoculum volume. Many PSF studies use relatively small amounts of conditioned soil mixed into a sterilized background, yet the extent to which dilution weakens or eliminates beneficial effects is rarely quantified, despite its importance for interpretation and applied use in restoration, agriculture, or forestry. Although soil chemistry and nutrients can influence plant–soil feedback under some conditions [17], many studies indicate that changes in the soil microbial community often dominate PSF outcomes and nutrient dynamics and that significant abiotic shifts unassociated with biotic shifts are uncommon [18,19,20,21].

Microbial dilution experiments show that reducing inoculum volume can alter microbial diversity, delay community establishment, and dampen functional outcomes, suggesting that PSFs may depend on exceeding a threshold density of soil biota [22,23]. Nonetheless, few studies explicitly test how inoculum volume interacts with other conditioning. As a result, it remains unclear whether an observed PSF effect will persist under dilution, or whether benefits depend on both soil volume and other factors.

Here, we investigated how a known conspecific positive soil conditioning, *Pinus radiata* D. Don, in New Zealand [8,24,25,26], varies at different inoculum volumes. By manipulating the proportion of conditioned soil added to a common background, we tested whether these benefits (in terms of increased biomass and decreased early mortality) decline with dilution of the soil inoculum. We hypothesized that these effects would weaken linearly as inoculum volume decreased.

## 2. Materials and Methods

### 2.1. Plant Material and Soil Collection

Cuttings of a *P. radiata* clone (referred to here as genotype G1) were collected from two-year-old mother plants and grown for one year in a commercial nursery before use in soil conditioning. New seedlings (of the same lineage as G1) were used to test the conditioned soil.

The soil used for the experiment was collected in Mawhera, New Zealand (42°29′ S 171°28′ E, New Zealand soil classification: Acidic-pedal Allophanic Brown Soils, US soil taxonomy: Hapludands). More information about the site can be found in [27]. The sample was processed aseptically and transported immediately after collection to the glasshouse, with potting completed within 24 h after collection (more information in Supplementary Materials).

### 2.2. Soil Conditioning Phase

**Figure 1:**
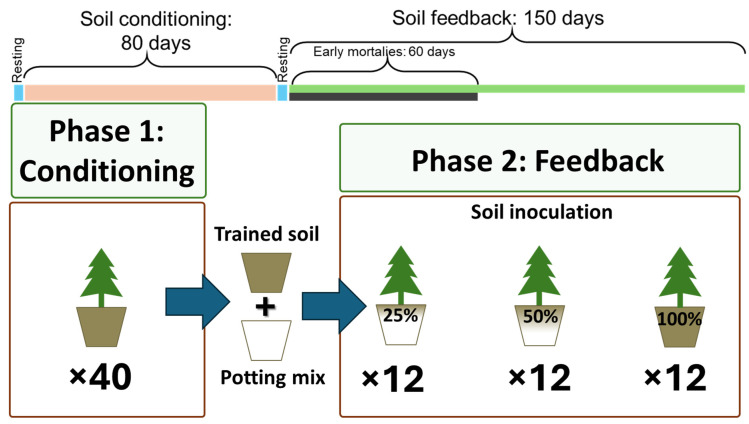
Experiment overview: 40 × G1 clones were used to condition the soil for 80 days, and 12 × G1 lineage seedlings were used to test each dilution for 150 days.

Soils were conditioned using G1 *P. radiata* seedlings, each in approximately 0.8–0.9 L of the field-collected Mawhera soil. Plants were grown in 1 L bleach-cleaned and water-rinsed pots in a glasshouse with the temperature maintained at 21 ± 2 °C. At the end of the conditioning phase, plants were removed, and soils were homogenized using a bleach-cleaned and clean water-rinsed cement mixer (Figure 1, Supplementary Material for more information). This soil-conditioning phase formed part of a broader experimental framework, but all steps relevant to the current feedback experiment are included here.

### 2.3. Soil Feedback Phase

Conditioned soils were tested with new G1-lineage *P. radiata* seedlings using three inoculation levels (100% soil, 50% soil + 50% inert media (pasteurized potting mix), 25% soil + 75% inert media (pasteurized potting mix)). Soil was pasteurised for 3 h at approximately 80 °C, then stirred with a clean rake (rake soaked in a 0.1% sodium hypochlorite solution for 10 min, then rinsed with clean water before each use), and then another 3 h at approximately 80 °C. The potting mix was a standard media, identical to that used by the nursery where the seedlings were grown and kept in stasis until transport, to simulate their previous conditions and minimize exposure to novel inputs (chemical and/or biological).

### 2.4. Sample Processing

Above-ground biomass was cut at the soil surface at apparent death (dominated by brown, drooping needles, see Supplementary Material for more information), all occurring within 60 days or at the conclusion of the experiment at 150 days. Below-ground biomass was gently washed clear of all soil in clean tap water. Total biomass (combined dried weight) was used in all analyses. All biomass was dried in paper bags at 50 °C, checked daily for mass loss, with final measurements made after 4–6 days, when the sample displayed constant mass as compared to the previous day; thus, constant mass was achieved at the time of measurement.

### 2.5. Data Analysis

Data was analyzed and visualized via R (version 4.2.2). Total dried biomass was initially analyzed using a linear mixed-effects model with dilution and harvest term (“early” meaning within 60 days, or “full” term indicating the entire feedback phase of 150 days) as fixed effects and replicate as a random intercept via the R package “glmmTMB” (version 1.1.13) [28].

To obtain the best model, terms and interactions were removed as appropriate via model simplification [29], and *p*-values for mixed effects models were calculated via the “lmerTest” package (version 3.1.3) [30]. Effects of dilution and harvest term (whether an early mortality or full-term growth) were tested using Type II Wald χ^2^ tests based on analysis of deviance. Because the variance associated with replicates was estimated as zero (singular fit), indicating no detectable among-replicate variation, replicates were removed, and analyses were conducted using fixed-effects models and R^2^ values using the R package “lme4” (version 1.1.35.5) [31]. The same model simplification technique and testing were used as with the mixed-effects models. For more information, see Supplementary Materials Table S1.

## 3. Results

### 3.1. Effects of Soil Dilution and Harvest Term on Seedling Biomass

**Figure 2:**
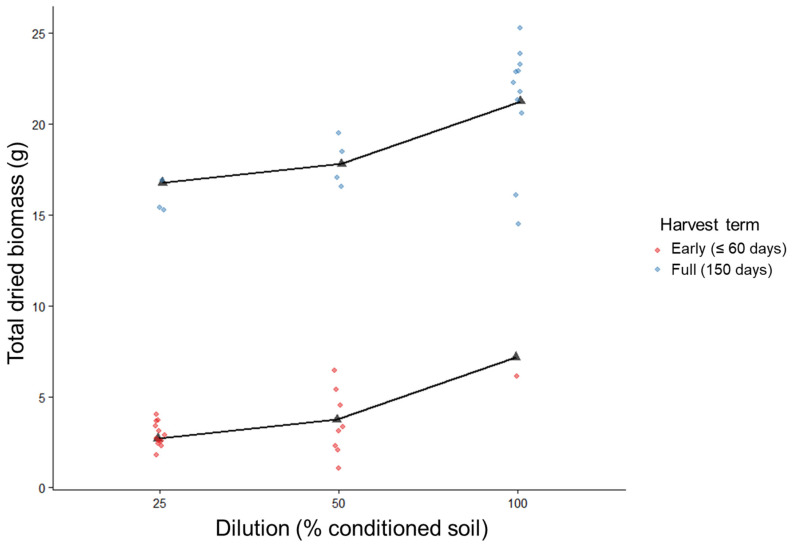
Model predictions (black triangles) with real data (circles) separated into early mortalities in red and full-term growth in blue, regression lines obtained from log-transformed model R^2^ = 0.95 and 0.94 for early and full-term biomass, respectively.

Soil dilution and harvest term both significantly influenced total dried biomass, while their interaction was not statistically significant in a linear model (Type II ANOVA; dilution: *F*_1,32_ = 16.65, *p* = 0.00028; harvest term: *F*_1,32_ = 230.82, *p* < 0.001; dilution × harvest term: *F*_1,32_ = 1.20, *p* = 0.28). Across harvest terms, increasing soil inoculum was associated with greater seedling biomass (Figure 2).

### 3.2. Nonlinear Response to Soil Dilution

Model comparison using Akaike Information Criterion (AIC) indicated that biomass responses to soil dilution were better described by a nonlinear relationship than a linear one. A model incorporating log-transformed biomass provided the best fit (AIC = 160.8), followed by the categorical dilution model (AIC = 163.9), whereas the linear dilution model was poorly supported (AIC = 174.0). Plotting log-transformed biomass against dilution revealed a clear pattern of diminishing returns with increasing soil volume, with steep gains from low to high inoculum levels and smaller gains from low to intermediate volumes (Figure 2). There were no significant trends in aboveground:belowground ratios, so only the combined dried biomass was used.

### 3.3. Early Mortality Patterns

Early mortality was strongly influenced by soil dilution (Figure 2). Reduced soil inoculum resulted in higher mortality, with the highest mortality observed at the lowest dilution levels. Only one seedling did not survive to full-term in the 100% soil treatment, indicating that adequate quantities of conditioned soil are required to buffer seedlings against stress during establishment.

### 3.4. Conclusions

Our results demonstrate that inoculum volume strongly influences positive plant–soil feedback outcomes, both seedling survival and biomass. In this system, soil conditioning created positive feedbacks but these benefits sharply declined as the proportion of conditioned soil decreased. At lower inoculum volumes, seedlings showed reduced biomass and increased early mortality. These findings align with previous work showing that soil legacies influence subsequent plant performance [2,4,13]. Although dilution with potting mix may have introduced minor artefacts, this approach was applied consistently across treatments and is unlikely to explain the observed patterns.

As the proportion of conditioned soil decreased, the growth benefits declined. This result highlights an important but rarely tested aspect of PSF experiments: the density of soil biota needed for functional outcomes. Although not explicitly tested here, dilution likely reduces microbial abundance and diversity, which disrupts key microbial interactions, consistent with microbial dilution-to-extinction theory [32]. Our results suggest that the absence of visible PSFs in some studies may reflect insufficient inoculum volume rather than a true lack of soil legacy effects. Biomass increased non-linearly, suggesting a potential threshold at 50% inoculum, although given our limited levels, this can only be inferred; additional research is needed to define this threshold more precisely.

The practical implications are clear. Many restoration, forestry, and management applications involve soil mixing, transport, or amendment at low volumes, potentially diluting soil legacies below functional levels. Future work should focus on identifying minimum effective inoculum thresholds, characterizing which microbial groups drive these effects, and determining how long soil-conditioned benefits persist in the environment.

## Data Availability

The data presented in this study are openly available in FigShare, reference number 31310875.

## Supplementary Materials

The following supporting information can be downloaded at: https://www.mdpi.com/article/10.3390/plants15050809/s1, Additional processing information, Table S1: Model information.
