# Supplementary material for: Volume Matters: Dilution of Soil Inoculum Reduces Positive Plant–Soil Feedback in Pinus radiata Seedlings
Source: Plants (Basel). 2026 Mar 6;15(5):809. doi: 10.3390/plants15050809 (PMC12987353; doi:10.3390/plants15050809)
Supplement: Supplementary file 1 [file plants-15-00809-s001.zip › plants-4178075-supplementary.pdf]

Supplementary Materials

Additional processing information:

Aseptic processing entails: use of new clean gloves and bags when handling, and clean equipment (shovels, pots, etc), that have been cleaned with 0.1% sodium hypochlorite solution and allowed to sit for 10 minutes followed by two times rinsed with clean water.

Cement mixer was run in 5 minute increments, until there were no visible clumps. After processed mixture removed, cement mixer was rinsed with 0.1% sodium hypochlorite solution and allowed to sit for 10 minutes followed by two times clean water rinses covering the sides and barrel in between different soils.

Pot location was fully randomized.

Apparent death was validated by dramatic drop in chlorophyll measurements. Chlorophyll was measured via Konica Minolta SPAD-502+ after a spectrophotometry calibration curve was created via a Campspec M501.

Supplementary Table: S1

**Model # : Equation**	**Compare (model numbers)**	***AIC***	***BIC***	***P***
**glmmTMB**				
Model1: log(total.dried.biomass) ~ dilution * harvest.term (as binary) + (1 | replicate)		165.9	178.6	
Model2: log(total.dried.biomass) ~ dilution + harvest.term (as binary) + (1 | replicate)	anova(1,2)	163.9	173.4	0.37
Model3: log(total.dried.biomass) ~ dilution (1 | replicate)	anova(2,3)	231.0	238.9	$2.3e^{-16}$
**lmer**				
Model1: log(total.dried.biomass) ~ dilution * harvest.term (as binary)		160.8	168.7	
Model2: log(total.dried.biomass) ~ dilution + harvest.term (as binary)		161.8	168.0	0.11
